# Supplementary material for: Interindividual Differences in In Vitro Human Intestinal Microbial Conversion of 3-Acetyl-DON and 15-Acetyl-DON
Source: Toxins (Basel). 2022 Mar 7;14(3):199. doi: 10.3390/toxins14030199 (PMC8953914; doi:10.3390/toxins14030199)
Supplement: Supplementary file 1 [file toxins-14-00199-s001.zip › toxins-1607649-supplementary.pdf]

toxins

MDPI

Supplementary Materials

Interindividual Differences in In Vitro Human Intestinal Microbial Conversion of 3-acetyl-DON and 15-acetyl-DON

Fangfang Li [1,†], Jing Jin [1,2,†], Ivonne M. C. M. Rietjens [2] and Fuguo Xing [1,*]

[1] Institute of Food Science and Technology, Chinese Academy of Agricultural Sciences/Key Laboratory of Agro-Products Quality and Safety Control in Storage and Transport Process, Ministry of Agriculture and Rural Affairs of P. R. China, 2 Yuanmingyuan West Road, Haidian District, Beijing 100193, China; l742240749@163.com (F.L.); jinjing@caas.cn (J.J.)
[2] Division of Toxicology, Wageningen University and Research, Stippeneng 4, 6708 WE Wageningen, The Netherlands; ivonne.rietjens@wur.nl
* Correspondence: xingfuguo@caas.cn
† These authors contribute equally to this work.

Table S1. Information for the individual fecal samples collected in this study.

Individual	Gender	Age
1	M	26
2	M	25
3	F	25
4	F	25
5	F	25
6	F	24
7	F	25
8	F	26
9	F	25
10	M	30
11	M	26
12	F	24
13	F	26
14	F	25
15	M	27
16	F	25
17	F	25
18	F	25
19	F	25
20	M	26
21	F	25
22	F	25
23	F	25
24	F	25
25	F	24

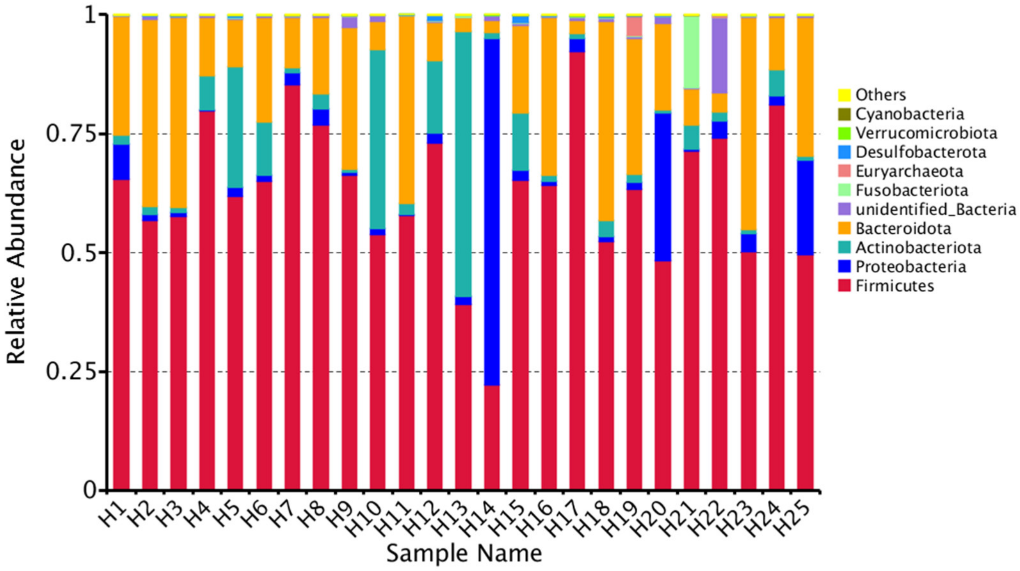

Figure S1. Analysis of the microbiome relative abundance of species at the phylum level in individual human samples.
